# Hepatitis C virus (HCV) infection in Africa: a review

**DOI:** 10.11604/pamj.2013.14.44.2199

**Published:** 2013-01-31

**Authors:** Mercy Jelagat Karoney, Abraham Mogisi Siika

**Affiliations:** 1 Moi University Clinical Research Center, Eldoret, Kenya; 2 Department of Medicine, School of Medicine, College of Health Sciences, Moi University, Eldoret Kenya

**Keywords:** Hepatitis C, prevalence, disease burden, treatment, prevention, Africa

## Abstract

Hepatitis C virus (HCV) is a viral pandemic and a leading cause of chronic liver disease. This review highlights the epidemiology and management of Hepatitis C in Africa. We searched for articles on medline using the terms, “Hepatitis C”, “Prevalence”, “Epidemiology”, “Africa” and “Treatment”. The bibliographies of the articles found were used to find other references. We included articles published after 1995 only. The data was summarized and presented in tables and figures. Africa has the highest WHO estimated regional HCV prevalence (5.3%). Egypt has the highest prevalence (17.5%) of HCV in the world. Genotypes commonly found in Africa are 1, 4 and 5. Genotype 3 is found in Egypt and parts of Central Africa. Blood transfusion is a major means of acquisition of HCV infection. While treatment with peginterferon and ribavirin is recommended for patients with chronic HCV, no data were found on their use in Africa. Neither were there any data on definitive management (liver transplantation) for those with end stage disease. Data on HCV infection in Africa are scarce. This suggests that hepatitis C is still a neglected disease in many countries. Limited data exist in literature on HCV in Africa.

## Background

Hepatitis C virus (HCV) is a RNA virus known to infect humans and chimpanzees, causing similar disease in these 2 species. HCV is most often transmitted parenterally but is also transmitted vertically and sexually [1]. HCV is up to 4 times more infectious than Human Immunodeficiency Virus (HIV). It also requires less exposure that HIV to cause infection [2].

HCV is a leading cause of chronic liver disease in the world [3, 4]. The World Health Organization (WHO) estimates that 170 million people are infected with HCV globally and 3-4 million new infections occur each year [5], making it one of the leading public health problems in the world. With a prevalence of 5.3% and an estimated 32 million people infected with HCV, Sub Saharan Africa has the highest burden of the disease in the world [6]. Other WHO regions with a high prevalence of HCV include Eastern Mediterranean (prevalence 4.6%) and Western Pacific (prevalence 3.9%). A summary of the regional burden of disease is provided in Table 1.

**Table 1:** Hepatitis C Virus: estimated prevalence and number infected; by WHO Region

WHO Region	Total Population (Millions)	Hepatitis C Virus prevalence rate (%)	Infected Population (Millions)
Africa	602	5.3	31.9
Americas	785	1.7	13.1
South-East Asia	1,500	2.15	32.3
Eastern Mediterranean	466	4.6	21.3
Europe	858	1.03	8.9
Western Pacific	1,600	3.9	62.2
**Total**	**5,811**	**3.1**	**169.7**

Despite its high prevalence and highly infectious nature, HCV remains under-diagnosed and underreported in Africa (with the exception of Egypt). Most of the available data on HCV in Africa are old and outdated. Because of such paucity in available data, little attention has been given to HCV in Africa. We therefore set out to review available medical literature on HCV in Africa with a view to determining the prevalence, disease burden and common transmission modes. In addition we draw attention to diagnosis, treatment and prevention of HCV.

## Methods

We searched medical literature in biomedical databases PUBMED, OVID and Google scholar using the following key words: “Hepatitis C”, “Prevalence”, “Epidemiology”, “Africa”, and “Treatment”. We limited the search to articles published in and after 1995. The bibliographies of the articles on hand were used to find other references. We also searched through indexes of major journals that publish on HCV.

Of the 600 articles we found only 49 were included in the final review. These were articles that had data on prevalence, transmission and disease burden of HCV in Africa; diagnosis and treatment guidelines. We did not find any articles on HCV treatment in Africa.

This was a Meta analysis that entailed systematic review of all articles in Africa with relevant HCV information. We gathered detailed data from relevant articles that met the criteria and organized them into a database. The studies were categorized by country and the variable of interest, prevalence, presented in tables and figures. We also gathered articles with guidelines on diagnosis, treatment and prevention of HCV and presented a summary of the findings.

## Current status of knowledge

### Disease burden and distribution

The estimated prevalence of HCV in Africa is 5.3% [7]. Egypt has the highest worldwide prevalence (17.5%). Egypt's unusually high prevalence is attributable to the history of unsterile injection equipment use for mass treatment of the general population with parenteral antischistosomal therapy (PAT) from the 1920s to the 1980s [8, 9]. The prevalence of HCV increases with age, with the highest rate being reported in the age group older than 40 years. No data were available on HCV morbidity and mortality in Africa. However, based on the general trends for most other diseases, it is possible that these indicators may be worse than the WHO reports of 75% of HCV-infected individuals developing chronic liver disease. Of those HCV-infected patients who develop chronic liver disease 1.6% progress to Hepatocellular carcinoma (HCC), a condition with a mortality rate >80%.[10].

### Transmission

The routes of transmission of HCV described in literature are: blood, blood products, tissue and organs; unsafe medical procedure; healthcare exposure e.g. needle stick injury [11]; intravenous drug use [12]; sexual transmission [13]; body piercings [14] and vertical transmission[15]. In Africa, only 19% of blood is screened for HCV (anti HCV antibodies). The main reason for this low screen rate is the prohibitive cost of the laboratory tests [16]. Also, inconsistent screening procedures for blood donors make blood transfusion a major means of acquisition of HCV infection. This is evidenced by a high HCV prevalence in sickle cell patients (17%) who have received multiple blood transfusions [17]. While reported prevalence of HCV in intravenous drug users in the developed world is as high as 80%, little is known about the prevalence of similar risk groups in Africa [18]. However, Madhava et al found drug use to be an uncommon means of HCV transmission in Africa [6]. While there is significant variation between countries, WHO estimates that in sub Saharan Africa, approximately 18% of injections are given with reused syringes or unsterilized needles thus increasing risk of transmission through unsafe injection practices [19]. Vertical transmission is low but significant in the setting of co-infection with HIV, a condition that is of pandemic proportions in Africa [20].

### Prevalence

The prevalence of HCV in the general population in Africa ranges between 0.1% and 17.5%, depending on the country. The countries with the highest prevalence include Egypt (17.5%), Cameroon (13.8%) and Burundi (11.3%). The countries with the lowest prevalence include Zambia, Kenya, Malawi and South Africa (all with a prevalence Table 2 gives details of the prevalence (and confidence intervals) of HCV in select African countries.

**Table 2:** Estimated HCV prevalence in general populations in African countries

Country	Sample size	HCV Prevalence (%) (CI)	Genotype
**Central Africa**			
Burundi	1184	11.3 (4.9 – 33.3)	4
Cameroon	6015	13.8(0.0 – 40.0)	4
CAR	709	2.4(0.0-6.1)	4
Chad	290	4.8(2.4-5.8)	4
Congo	0	(2.5-9.2)	4
DRC	2572	5.5(4.3-6.6)	4
Equatorial Guinea	2042	1.7(1.7-1.7)	4
Gabon	1597	9.2(6.5-16.5)	4
Rwanda	610	4.1(0.9-17.0)	4
Sudan	865	2.8(1.5-3.2)	4
Uganda	881	6.6(0.0-14.2)	4
**Total**	16765	6.0(0.0-40.0)	4
**West Africa**			
Benin	1110	1.6(0.0-4.0)	1-3
Burkina Faso	965	4.9(2.2-8.3)	1-3
Cote d'Ivoire	429	3.3(3.3-8.2)	1-3
Gambia	212	2.4(2.4-2.4)	1-3
Ghana	5033	1.7(0.1-5.4)	1-3
Guinea	2050	5.5(0.8-8.7)	1-3
Mauritania	349	1.1(1.1-1.1)	1-3
Niger	2327	1.8(0.0-7.6)	1-3
Nigeria	669	2.1(0.0-5.8)	1-3
Senegal	352	2.2(0.0-7.3)	1-3
Togo	478	3.9(1.3-6.1)	1-3
Total	13974	2.4(0.0-8.7)	1-3
**South and east Africa**			
Eritrea	323	1.9(0.0-6.0)	1-3
Ethiopia	2080	1.9(0.6-3.4)	1-3
Kenya	1567	0.9(0.0-1.0)	1-3
Madagascar	1564	2.1(1.2-3.3)	
Malawi	140	0.7(0.7-0.7)	
Mozambique	536	2.8(2.1-3.2)	
Somalia	2203	1.5(0.0-7.0)	
South Africa	68931	0.1(0.0-3.5)	5
Swaziland	194	1.5(1.5-1.5)	
Tanzania	2188	3.2(0.5-8.6)	5
Zambia	583	0.2(0.0-0.3)	5
Zimbabwe	579	2.0(0.2-7.7)	
**S & E Africa total**	80888	1.6(0.0-8.6)	5
* **North Africa**			
Egypt		17.5(13-22)	4
Sudan		3	4
Libya		1.2	4/1
Tunisia		0.55(0.4-0.7)	1b
Algeria		1.8	NA
Mauritania		1.8	NA
Morocco		7.7	1b

* no available data on sample size and confidence interval for some countries

### Risk groups

High risk populations include: Intravenous drug users; HIV-infected; patients on hemodialysis; patients with history of blood transfusions or organ transplantation; health care workers after needle stick injuries; children born to HCV infected mothers. Also, sexually active adults with multiple partners have higher prevalence rates. Available data on HCV reveal high prevalence in patients with hepatocellular carcinoma or chronic liver disease: (Burundi; 55%, Rwanda; 45.7%) and sexually transmitted diseases (Ethiopia; 38.2%). Countries with low HCV prevalence in high-risk groups include Zimbabwe (1.3%) and Kenya (1.7%) [6].

### Genotypes

There are 11 HCV genotypes: 1-11, with many subtypes: a, b, c, and about 100 different strains: 1,2,3 based on the sequence of the HCV genome [3]. Genotypes 1-3 are widely distributed globally, with genotypes 1a and 1 b accounting for 60% of infections worldwide. Genotype 4 is characteristic for the Middle East, Egypt and Central Africa. Genotype 5 is almost exclusively found in South Africa [1, 21]. More information on genotype distribution is available in Table 2.

### Disease progression

Few data are available on natural history and progression of HCV infection in Africa. However studies done on African Americans show higher rates of chronic HCV infection compared to whites [22]. Acute infections and less advanced stages of chronic disease are clinically silent [23] and only about half of the viremic patients exhibit elevated Alanine Aminotransferase (ALT) activity [24]. HCV is often first diagnosed in late stage when the therapeutic options are already limited. Due to slow and silent onset, many patients are unaware of their infection and at least 40% cases remain undetected [2].

Chronic hepatitis C is difficult to assess, because it is frequently subclinical. Patients with chronic hepatitis C are at risk of cirrhosis and hepatocellular carcinoma and their contacts at risk of acquiring the infection through exposure to the virus [25–28]. The risk of developing cirrhosis ranges from 5% to 25% over periods of 25 to 30 years [29–31].

### Diagnosis

HCV testing is recommended among persons with high risk of getting infected and patients with unexplained high ALT levels [1]. Highly sensitive and specific rapid tests for diagnosis of HCV are available. HCV RNA can be detected in the blood using amplification techniques such as polymerase chain reaction (PCR) or transcription-mediated amplification (TMA) [32]. Quantitative HCV RNA should be determined before initiating treatment. Follow-up HCV RNA is useful in monitoring success of HCV treatment [33].

Although genotyping does not predict the outcome of infection [25, 27, 28], it is useful in predicting the likelihood of treatment response and determines the duration of treatment in many cases as discussed in the Treatment section below.

### Treatment indications

All patients with chronic hepatitis C infection should be considered potential candidates for drug therapy [34]. Treatment is recommended for patients who are at risk of developing cirrhosis, generally defined by a measurable hepatitis C RNA level and liver biopsy showing portal or ridging fibrosis along with moderate inflammation and necrosis[35]. Treatment is also recommended for patients with elevated serum ALT levels who meet the following criteria [36]:

1. Age >18 years
2. Positive HCV antibody and serum HCV RNA test results
3. Compensated liver disease (e.g., no hepatic encephalopathy or ascites)
4. Acceptable hematologic and biochemical indices (hemoglobin at least 13 g/dL for men and 12 g/dL for women; neutrophil count >1500/mm3, serum creatinine < 1.5 mg/dL)
5. Willingness to be treated and to adhere to treatment requirements
6. No contraindications for treatment

A pretreatment liver biopsy is not mandatory but may be helpful in patients with normal transaminase levels, particularly those with a history of alcohol dependence, in whom little correlation may exist between liver enzyme levels and histologic findings [37].

### Recommended treatment regimens

Spontaneous resolution of hepatitis C virus is common and waiting 2-4 months before initiation of therapy is recommended [37]. The objective of therapy is to eradicate the virus and prevent potential complications from chronic HCV infection. If detected early, progression of chronic hepatitis to severe liver disease can be prevented in 54-63% of patients through antiviral treatment [25–28]. Efficacy of treatment is assessed by measuring Hepatitis C RNA viral load. The goal is to achieve a Sustained Virological Rate (SVR), defined by the continued absence of hepatitis C RNA 6 months after the completion of treatment [35, 37]. Treatment for chronic HCV infection has evolved from interferon monotherapy, which results in an SVR of 10 to 20% [38] to combination therapy with interferon plus ribavirin, which is associated with a higher SVR rate of nearly 40% [39–41].

The duration of standard interferon plus ribavirin therapy has been based on the viral genotype and the pre-treatment viral load [42]. The SVR rates for patients infected with genotype 2 or 3 are essentially the same for 24 and 48 weeks of therapy, showing no benefit for the longer course of therapy [33, 40]. For patients infected with genotype 1 isolates, 48 weeks of interferon plus ribavirin therapy is recommended for those with a high viral load (>800,000 IU/ml) and only 24 weeks of therapy for patients with those with a low pre-treatment viral load [43, 44]. Table 3 shows a summary of the recommended treatment regimens. Despite the improved results achieved with the addition of ribavirin to PEG-IFN, the current available therapies for chronic HCV infection are effective in fewer than 50% of patients with HCV genotype 1. Protease inhibitors (PI) used in conjunction with pegylated interferon and ribavirin is becoming the new standard of care for the treatment of chronic HCV infection [37]. In HCV genotype 1-infected patients without HIV, addition of an HCV NS3/4A PI boceprevir or telaprevir to PegIFN/RBV significantly improves the rate of sustained virologic response (SVR) [45, 46].

**Table 3:** Recommended treatment regimens for hepatitis C virus infection

	Medication	Dose	Duration
Acute infection	Interferon (IFN)	6MU IM/SC x3/Week	36 weeks
	PEG Interferon	180 mcg weekly	48 weeks
Chronic infection	Interferon	3MU IM/SC x3/Week	24months
	Ribavirin	800-1200mg PO BD	24-48 weeks

Liver transplant is the only therapeutic option for patients with end stage liver disease [23, 47]. The drugs used to treat Hepatitis C cost approximately $30,000 for 48 weeks. The cost of treating side effects of these drugs further increase the cost of treating hepatitis C. Future hepatitis C drugs are expected to be more expensive [48].

### Prevention strategies

Primary prevention activities include: screening and testing of blood, plasma, tissue, organ and semen donors; virus inactivation of plasma derived products; risk reduction counseling services and implementation of infection-control practices. Secondary prevention activities includes identification and testing of persons at risk and management of infected persons [1].

## Conclusion

The paucity of available information indicates that hepatitis C is still a neglected disease in many countries. However, from the scanty data presented, there is no doubt that HCV is a major health problem that requires greater attention in Africa. With availability of effective therapies against HCV, physicians, researchers and health care decision makers need to improve efforts in diagnosis, management and prevention of HCV in Africa. The relatively high cost of treatment enforces the need for a systematic approach for this condition so that resources are used most effectively.
